# Willingness of general dental practices in South East London to engage with research

**DOI:** 10.1017/S1463423618000944

**Published:** 2019-07-24

**Authors:** Marwah Afeef, Caillin Redican, Eduardo Bernabé

**Affiliations:** 1 Faculty of Dentistry, Oral & Craniofacial Sciences King’s College London, London, UK; 2 South East London Health Education England, London, UK

**Keywords:** dental health services, general dental practice, primary health care, research

## Abstract

This study assessed the willingness of general dental practices (GDPs) to participate in research. All 263 GDPs in South East London that provide dental care under National Health Service (NHS) contracts were invited. The survey instrument was adapted from previous studies and piloted before administration. Geographical factors and practice characteristics associated with willingness to participate in research were explored in logistic regression models. A total of 77 responses were received (response rate: 29%). Of them, 40 (53%) expressed interest in being involved in primary care research. They saw their main role as collecting data and facilitating access to patients. Time, bureaucracy and lack of energy were the main reasons behind a decision not to engage with research. Those spending more time in NHS services were more likely to be willing to participate in research. Other possible indicators were single-handed GDPs, participation in the dental foundation training programme and location in more affluent areas.

## Introduction

Secondary care and academic settings are the default options adopted by researchers to generate evidence-based knowledge (Kidd *et al*., 2014). However, results from secondary care research are not directly applicable to patients seen in primary care because diagnostic criteria and thresholds are different between patients who are at early or late stage of the disease process (Furler *et al*., 2008; Fox *et al*., 2014). Thus, primary care settings are in need for relevant evidence that meets the local population needs (Fox *et al*., 2014; Heasman *et al*., 2015). Research networks are the key to sustain and strengthen research capacity in primary care (Vezyridis and Timmons, 2016; Koskela, 2017). Unlike those in medicine, research networks in primary dental care are in an early stage of development (Kay *et al*., 2003; Heasman *et al*., 2015; Hare *et al*., 2018). The purpose of this study was to assess the willingness of general dental practices (GDPs) in South East London to participate in collaborative research with academics.

## Methods

### Study population

All 263 National Health Service (NHS) GDPs within inner (four boroughs: Lambeth, Southwark, Lewisham and Greenwich) and outer South East London (three boroughs: Bexley, Bromley and Croydon) that provide dental care under NHS contracts were invited to participate in this survey. The list of GDPs was retrieved from the online NHS primary dental care services locator (https://www.nhs.uk/service-search) in March 2018. The postcodes included within the geographical boundaries of each selected borough were confirmed using the Department for Communities and Local Government postcode lookup website (http://imd-by-postcode.opendatacommunities.org/). The study protocol was registered as a minimal ethical risk project with King’s College London Research Ethics Committee (reference: MR/17/18-330).

### Data collection

Data were collected through a postal questionnaire. The survey instrument, adapted from previous surveys (Bedos and Allison, 2004; Palmer and Grieveson, 2005; Stout *et al*., 2014), comprised 16 questions organised under four sections. The first section collected participants’ opinions about the value and availability of research results. The second section collected information on past research experience, including questions on previous roles held, funding and training. The third section gathered information on the willingness of GDPs to participate in dental research. In case of a positive answer, a follow-up question enquired about the roles in which they would like to get involved. In case of a negative answer, a subsequent open-ended question was asked about the reasons behind that decision. The last section of the instrument collected information on GDPs’ characteristics. The cover letter and questionnaire were piloted for content and face validity among five dentists working in primary care (two owners, one owner/academic and two associates) with whom the division of population and patient health has an ongoing relationship. The instrument was amended based on the feedback provided. Piloting also informed how much time was needed to complete the questionnaire so that respondents were correctly informed in the cover letter.

Questionnaires were posted to all GDPs in sealed envelopes, containing a cover letter, the questionnaire and a prepaid envelope for GDPs to send responses back. The cover letter was addressed to the owner of the practice and informed them about the study and what information was required from them. All questionnaires were coded before posting so that GDPs could be identified for further contact if they provided a positive reply. To enhance response rate, a reminder was sent out to all GDPs for which a response was not received after two weeks. Based on their postcode, GDPs were assigned to one of the 2015 English Index of Multiple Deprivation deciles (Smith *et al*., 2015).

### Data analysis

Analysis was performed using the Statistics Package for Social Sciences for Windows (IBM Corp., Armonk, NY, USA). GDPs were the unit of analysis. We first described the characteristics of participating GDPs and summarise GDPs’ opinions about research value and availability. Thereafter, we explored the association of geographical factors (location and area deprivation), practice characteristics (services provided, time spent in NHS services, type of practice and practice size) and respondents’ opinion about research value and availability with willingness of GDPs to participate in research using logistic regression. Odds ratios (OR) were reported as the measure of association (Hosmer *et al*., 2013).

## Results

A total of 77 responses were received, representing a response rate of 29%. No differences between participating and non-participating GDPs were found according to location or deprivation level. Table 1 presents the characteristics of participating GDPs. Most questionnaires (75%) were completed by the owner/principal of the GDP. Most GDPs provided mixed services, with the median proportion of time spent on NHS services being 80% (inter-quartile range: 55; range: 0–100). Most GDPs were group practices, with a median of four (inter-quartile range: 2; range: 1–0) dentists per GDP.

**Table 1:** Characteristics of participating general dental practices

Characteristics	*n/N*	%
Area		
Inner South East London	36/66	54.6
Outer South East London	30/66	45.4
Deprivation level		
1st–3rd decile (most deprived)	24/63	38.1
4th–7th decile	29/63	46.0
8th–10th decile (most affluent)	10/63	15.9
Services provided		
NHS only	15/75	20.0
Mixed (NHS and private)	60/75	80.0
Type of practice		
Solo	8/73	11.0
Group	65/73	89.0
Practice size		
Single-handed	9/64	14.1
2–4 dentists	31/64	48.4
5 or more dentists	24/64	37.5
Dental foundation training *site*		
No	51/77	66.2
Yes	26/77	33.8

In terms of their views on research value and availability, 53% of respondents stated that the results of dental research were not easily available to them. Professional journals were the most common source of information (78%), followed by internet resources (39%). Furthermore, 74% reported that results from dental research have changed their clinical behaviour whereas 47% reported that dental research has had quite a big or a very big effect on the oral health of the British population. Examples with the greatest impact on clinical practice were non-surgical treatment of periodontal disease, sensitivity toothpaste, dentine bonding agents and frequency of recall intervals. Examples of research impact on people’s oral health were the role of sugars on dental caries, fluoride efficacy and harms of smoking.

Only 18% of respondents reported previous research involvement. Among them, 62% reported having received research training. As for funding, only 15% had received funds for either staff, costs and their time. The most common role reported was collecting information (69%), followed by analysing data and writing reports (46%), and design and management of research projects (31%).

A total of 40 GDPs (53%) expressed interest in being involved in research. They saw their main role as collecting data and facilitating access to patients (87%) as well as being part of the planning and discussion process (46%). Time, NHS bureaucracy and lack of energy were the main reasons behind a decision not to engage in research. Some factors were associated with GDPs willingness to engage in research (Table 2). The amount of time spent providing NHS services was positively associated with willingness to participate in research (OR: 1.02; 95% CI: 1.01–1.03 per unit increase in the time spent in NHS care). Solo GDPs (63% versus 53%), those involved in dental foundation training (DFT) (60% versus 50%) and those in the most affluent areas (60% versus 54%), also seemed more likely to be willing to participate in research than their respective counterparts. However, these differences were not statistically significant. Other factors were not associated with willingness to participate in research.

**Table 2:** Factors associated with willingness of general dental practices to participate in research

	Willingness to participate in research				
Factors	*n/N*	%	OR^a^	[95% CI]	*P-*value
Area					
Inner South East London	20/35	57.1	1.00	[Reference]	
Outer South East London	15/29	51.7	0.80	[0.30–0.16]	0.665
Deprivation level					
1st–3rd decile (most deprived)	13/24	54.2	1.00	[Reference]	
4th–7th decile	15/28	53.6	0.98	[0.33–0.91]	0.966
8th–10th decile (most affluent)	6/10	60.0	1.27	[0.28–0.68]	0.755
Services provided					
NHS only	8/15	53.3	1.00	[Reference]	
Mixed (NHS and private)	32/59	54.2	1.04	[0.33–0.23]	0.950
Time spent on NHS services	–	–	1.02	[1.01–0.03]	0.032
Type of practice					
Solo	5/8	62.5	1.00	[Reference]	
Group	34/64	53.1	0.68	[0.15–0.08]	0.617
Dental foundation training site					
No	25/50	50.0	1.00	[Reference]	
Yes	15/25	60.0	1.50	[0.57–0.97]	0.414
Practice size					
Single-handed	5/9	55.6	1.00	[Reference]	
2–4 dentists	16/30	53.3	0.91	[0.20–0.09]	0.907
5 or more dentists	14/24	58.3	1.12	[0.24–0.25]	0.886
Previous research involvement					
No	33/60	55.0	1.00	[Reference]	
Yes	7/13	53.9	0.95	[0.29–0.18]	0.940

a Simple binary logistic regression was fitted. Odds ratios (OR) are reported.

## Discussion

This study shows that more than half of respondents were willing to be involved in research. Respondents saw themselves involved mainly as data collectors and facilitators (ie, granting access to patients and dental records), although they would also like to be part of the planning and discussion process. This result agrees with a previous UK study where most respondents reported to have been involved as data collectors (Palmer and Grieveson, 2005). It is interesting to note that a previous study to test the feasibility of creating and maintaining a research network of GDPs revealed that dentists collecting data found the topics not very useful to their clinical practice (Makansi *et al*., 2010). Therefore, caution must be taken, and dental practitioners must be encouraged to take an active role in putting forward research topics relevant to their practice and patients.

GDPs with no interest to participate in research revealed lack of time as the main reason behind their decision. This is consistent with previous studies in which time constraints were identified as the major barrier to participation (Hopper *et al*., 2011; Stout *et al*., 2014; Heasman *et al*., 2015). GDPs are busy settings with many competing priorities. Therefore, some form of provision for loss of earnings or protected time to allow for periods away from service and training could help addressing this barrier (Kay *et al*., 2003). Although some GDPs preferred not to be actively involved in research, our findings show that most valued research considered it beneficial to practice and people.

Our study also revealed that time spent on NHS care was associated with willingness to participate in research. The increase in willingness to participate in research according to the time the GDP spent on NHS services suggests that longer contact with NHS patients induces greater awareness of the local population needs and drives dentists to seek solutions to address those needs. The other possible indicators to participate in research were solo GDPs, involvement in DFT and area deprivation. Solo GDPs may feel more in control of resources and time available. GDPs involved as DFT sites might be more likely to participate in research because of their positive experience with another external organisation such as Health Education England. DFT sites offer an opportunity to conduct research with junior staff (ie, foundation dentists) who may want to keep in touch with the academic environment they have just left. In addition, most GDPs with interest to participate in research were from more affluent areas. Given the well-known links between socioeconomic position, dental behaviours and oral health status (Thomson, 2012; Sabbah *et al*., 2015; Steele *et al*., 2015), this finding is somewhat worrisome because it suggests people with the greatest need (ie, patients in more deprived areas) will not benefit directly from the participation of their local GDPs in clinical research. If this finding is confirmed, targeted recruitment strategies might be needed to ensure participation of GDPs from deprived areas in clinical trials.

The present findings have some implications. There is certainly an interest from GDPs to be part of local research network with academics. Our findings help characterise the type of GDPs more likely to participate in research. Further contact with GDPs willing to engage in research may open the door to find common research topics and facilitate research in primary dental care. We expect that once a primary dental care research network is established locally it will encourage more GDPs to join. Furthermore, barriers to engage in research should be addressed to increase participation (Kay *et al*., 2003; Hare *et al*., 2018). For GDPs willing to participate, evaluation of research capability/resources and formal research training are the next steps to consolidate a local primary care research network and carry out clinical research in primary settings.

Limitations of this study include the low response rate and small sample size. Low response rates are usually expected from postal surveys, even when reminders are used (Bowling, 2005; Cottrell *et al*., 2015). However, our study response rate was in line with rates achieved in previous similar studies (Bedos and Allison, 2004; Palmer and Grieveson, 2005; Stout *et al*., 2014). Although other methods of questionnaire administration could have been used (ie, phone interviews or email surveys), they were initially disregarded as either intrusive or unfeasible (no access to list of NHS email addresses for instance). The fact only 77 responses were obtained limited our ability to test for factors associated with willingness to participate in research and probably identifying more significant associations. Therefore, the results of this study do not necessarily speak for all the GDPs with NHS contracts within the area of South East London.

In conclusion, this study shows that more than half of GDPs in South East London who responded to our postal questionnaire want to engage in research with academics. Time, NHS bureaucracy and lack of energy were the main barriers to engage in research. Those spending more time in NHS services were more likely to be willing to participate in research. Other possible indicators were single-handed GDPs, participation in the DFT programme and location in more affluent areas.
